# The CO-MAsk Approach: Tips for Fostering Mask Use Among Older Adults During the COVID-19 Pandemic

**DOI:** 10.5964/ejop.6815

**Published:** 2021-11-30

**Authors:** Marina Maffoni, Valeria Torlaschi, Paola Gabanelli, Paola Abelli, Antonia Pierobon

**Affiliations:** 1 Istituti Clinici Scientifici Maugeri IRCCS, Psychology Unit of Montescano Institute, Montescano (PV), Italy; 2 Istituti Clinici Scientifici Maugeri IRCCS, Psychology Unit of Pavia Institute, Pavia (PV), Italy; 3 Istituti Clinici Scientifici Maugeri, Health Administration of Montescano Institute, Montescano (PV), Italy

**Keywords:** COVID-19, older adults, face mask, adherence, psychoeducational

## Abstract

Face masks are effective at limiting contagion of the coronavirus. However, adherence to face mask use among the older adult population is often unsatisfactory due to cognitive impairment, misconceptions, and difficulty in retrieving face masks. This brief note provides healthcare professionals with simple suggestions about how to improve face mask adoption in the older adults, in particular if they suffer from mild cognitive impairment. Thus, clinical reflections and psychoeducational suggestions are summarized into a simple mental roadmap. Specifically, the CO-MAsk approach underlines the necessity to consider the following factors: Cognition (possible cognitive impairment), Occasions (real chances to access correct information and proper protection equipment), Motivation (individual motivation towards sanitary prescriptions) and Assumptions (personal beliefs and understandings). Possible obstacles and practical suggestions for are also discussed. It is of paramount importance that healthcare professionals pay attention to emotional, cognitive and psychological aspects to effectively improve the face masks adherence among older adults, specifically when cognitive decline is present.

*“Woe to him who doesn't know how to wear his mask”.*

— Pirandello (1952)

This statement, written by Luigi Pirandello, one of the biggest Italian dramatists, has never been more relevant and up-to-date than during the current coronavirus disease (COVID-19) pandemic. Indeed, the use of this personal protective equipment impacts not only on patients’ health, but also on the entire healthcare systems with relevant consequences on both political and social levels.

The face mask underwent various transformations during the history of humankind and, nowadays, its adoption has become one of the main topics in the ongoing crisis (Goh et al., 2020). This personal protection equipment is extremely relevant for frontline healthcare professionals who are more exposed to the risk of contracting the COVID-19 infection. The massive use of face masks among the population has been the subject of debate mainly due to the shortage of this equipment at the beginning of the pandemic outbreak (Goh et al., 2020; World Health Organization, 2020). Nevertheless, the scientific community is increasingly encouraging the universal donning of the face mask as it is considered a cheap and feasible measure to limit the infections of COVID-19, as well as a way to discourage possible discrimination of individuals obliged to wear this protection because they are more exposed to contagion than others (Goh et al., 2020; Li et al., 2020).

Indeed, being that the new coronavirus is a respiratory infection, facemask use may be a satisfactory non-pharmaceutical intervention to prevent the spread of the virus, thereby lower the risk of infection while reducing healthcare costs. In this regard, the recent history provided relevant examples. During the last Severe Acute Respiratory Syndrome (SARS) virus epidemic, it has been demonstrated that the human-to-human transmission could be reduced of 68% by wearing a face mask alone (Jefferson et al., 2020). Moreover, a recent modelling simulation indicated that wearing a face mask may effectively contribute to flatten the epidemic curve of the COVID-19 pandemic (Li et al., 2020).

Considering the increasing healthcare costs and the life-threatening risks of the ongoing pandemic for a certain part of the population, the use of face masks turns to be not only an ethical and moral imperative, but also a social and policy urgency which cannot be neglected. These considerations should promote the adoption of a novel mindset describing this personal protection equipment not only as a mere medical prescription, rather as the “new normal” and a shared social practice (van der Westhuizen et al., 2020).

This sounds particularly relevant for older adults who are more at risk of undergoing threatening consequences from a possible COVID-19 disease or other infections. On this matter, the clinical experience and the most recent literature demonstrated that the new coronavirus may be particularly deadly to those who are in advanced age (Liu et al., 2020). However, the use of this personal protection equipment cannot be taken for granted in the older adult population, in particular when mild cognitive decline is ongoing. Indeed, if wearing a mask is uncomfortable for the general population, it turns to be even more challenging for people who are not able to fully understand what is happening and what is the reason for donning face masks. This individual protection equipment also hides most of the face, posing difficulties in the recognition of people and in the transmission of emotions. Furthermore, it has to be noted that, in general, the older adult population’s adherence to medication and behavioral prescriptions is still a challenging and thorny issue regardless of the specific medical condition considered (Costa et al., 2017). In this vein, this paper aims to provide simple formal and informal caregivers with suggestions about how to improve face mask adoption in the older population, in particular when mild cognitive impairment is present.

## CO-MAsk Approach: A Theoretical Model to Overcome the Problem

**Figure 1:**
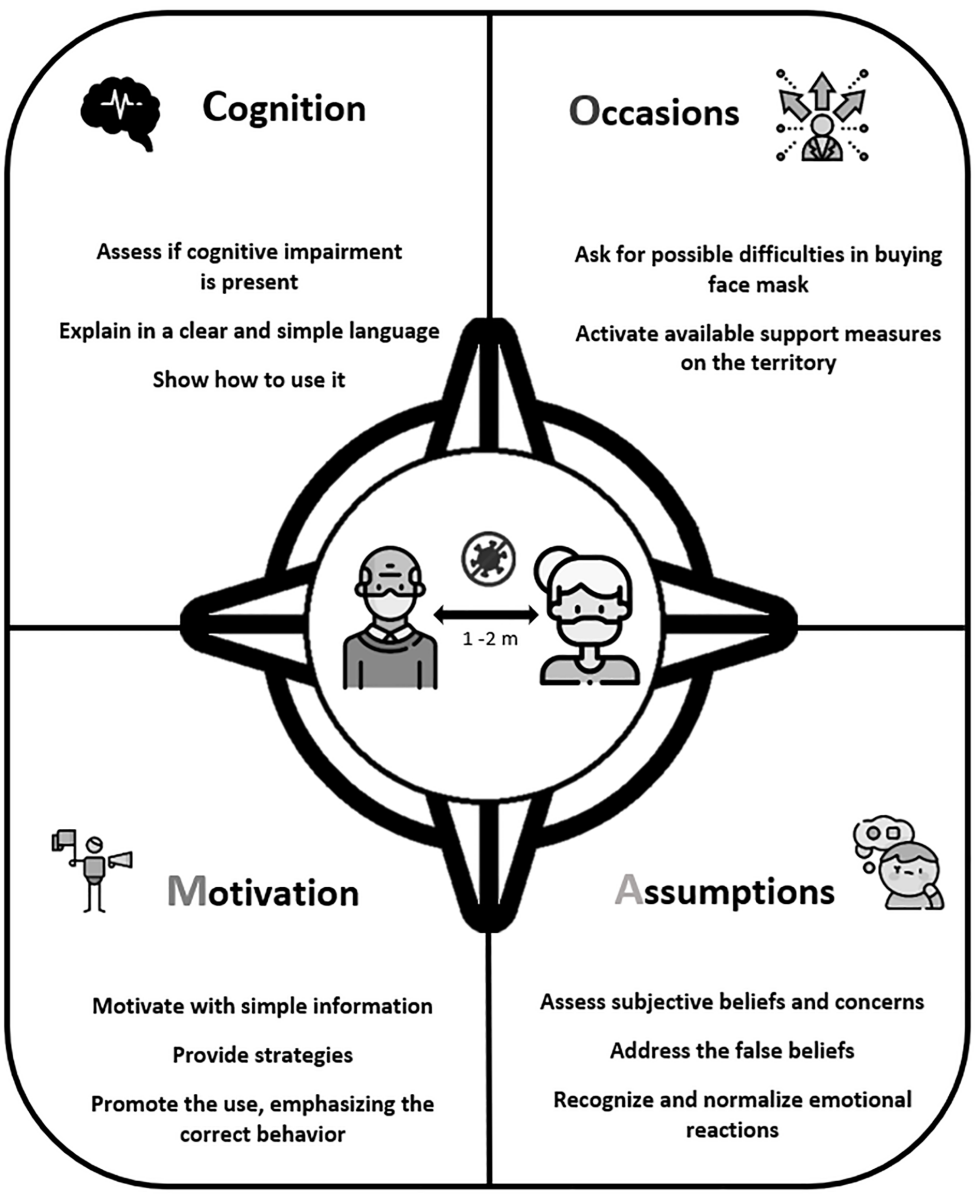
CO-MAsk – Approach: Foster Adherence to the Use of Face Masks Among Older Adults *Note.* The educational poster in english language (see the italian version in Supplementary Materials). Created by Istituti Clinici Scientifici Maugeri IRCCS, Psychology Unit of Montescano Institute (PV), Italy. Free icons by www.flaticon.com.

Manifold factors may prevent the effective and proper use of face masks among older adults. Healthcare professionals, healthcare administration and informal caregivers are therefore requested to develop strategies and approaches to foster the use of this personal protection equipment in this part of population. For this reason, we propose the *CO-MAsk approach* to highlight all the sensitive issues to consider when dealing with older adults, specifically if they are affected by mild cognitive impairment (See Table 1). An educational poster, which summarizes this approach, has been developed too (Figure 1). The term CO-MAsk is the acronym of the following pivotal aspects: Cognition, Occasions, Motivation and Assumptions.

**Table 1:** The CO-MAsk Approach for Fostering Mask Use Among Older Adults

CO-MAsk approach	Possible hindrance	Suggestion
**C**ognition	Is she/he aware of the current sanitary condition? Does she/he understand why to wear a face mask? Does she/he understand how correctly wear a face mask? Does she/he remember to wear a face mask?	Explain in a clear and simple language as far as possible considering the severity of the cognitive impairment. To show how to wear a face mask To reassure and calm the individual To consider other forms of protection in case of cognitive impairment (safer environments)
**O**ccasions	Does she/he have access to correct information? Does she/he have access to face masks? (physical and cognitive impairment, distance of shops and pharmacies, financial issues)	To ask for possible difficulties in buying face mask To activate all available support measures on the territory to help the individual
**M**otivation	Is she/he motivated to wear a face mask? Does she/he know the reasons for wearing a face mask?	To provide simple and clear information To provide strategies for wearing a mask
**A**ssumptions	Has she/he false misconceptions regarding the face mask? Is she/he scared by face mask?	To assess the individual beliefs and concerns To provide simple and clear information in response to false beliefs To recognize and normalize emotional reactions responses and to reassure

Firstly, *Cognition* refers to the presence of possible cognitive impairment which, even not being so severe, may prevent the individual from being fully aware of the actual sanitary condition and of the necessity to wear personal protection equipment. Literature has already underlined that cognitive impairment may be a strong hindrance to the correct adherence to medication and behavioral prescriptions as the individuals become unable to remember and manage drugs and medical devices by themselves (Maffoni & Giardini, 2017; Giardini et al., 2018; Maffoni et al., 2020; Maffoni et al., 2021). Thus, ignoring the reason of donning such an uncomfortable device, the person may instinctively throw away the face mask or being unable to correctly follow safeguarding procedures (Porcari et al., 2020). Thereby, it is suggested to not provide the mask as something imposed and mandatory, rather to try to explain with simple words the reasons under the necessity to wear a mask in accordance with the limits imposed by the presence of mild cognitive impairment. Moreover, showing how to wear the face mask may be also useful as it provides people to adequate strategies and example to manage this personal protective equipment. Overall, it is still an open question how to effectively address neurocognitive disorders which pose more vulnerable people to higher risk of contagion (Devita et al., 2021). Perhaps, in the daily clinical practice, it is necessary to give more importance to the context, creating safe and more protected environments. Likewise, reassuring and calming down the older person is essential to cope with the individual’s fears despite her/his understanding.

Secondly, it should be considered the actual *Occasions* a person may have. This aspect is particularly crucial to assure older adult’s adherence to face mask in phase of discharge. The term “occasion” means that it is important to pay attention to the real chances of accessing correct information and proper protection equipment (Porcari et al., 2020). For instance, older people may be affected by cognitive and/or physical impairments and not being able to go out from their home in order to reach pharmacies and other shops for buying face masks. These facilities may be also very distant, in particular in rural areas. In this regard, literature reported that older adults cope with manifold hindrances preventing their ability to access to health services (Horton & Johnson, 2010), and this issue is even more relevant with the increasing of age (Iecovich & Carmel, 2009). For instance, obstacles may include lack of transportation, lack of family and social support, complex health care procedures and poor communication with healthcare professionals regarding available options (Horton & Johnson, 2010). The financial aspect should be also considered as a possible hindrance, especially where the healthcare system is based on health assurances which are not always affordable to everyone (Horton & Johnson, 2010). All these aspects are more and more crucial in the time of the current pandemic (Mesa Vieira et al., 2020), but also in possible future sanitary challanges. Therefore, it is pivotal to ask about possible impediments in the provision of personal protection equipment and to activate all available support measures (e.g., social and sanitary assistance services). This is crucial for not letting the person be alone when at home.

Thirdly, another aspect to consider is *Motivation* which is a crucial element for fostering adherence as it positively predisposes the individual towards prescriptions (Martin & DiMatteo, 2013). The huge amount of information may overwhelm and create confusion to the person who, in turn, may be not so motivated in wearing the face mask. This is even more critical for people suffering from cognitive disorders (Devita et al., 2021; Porcari et al., 2020). If people do not fully understand the reasons to wear a face mask, they do not have any motivation to use it as this personal protection equipment may be considered only an unusual and uncomfortable device or even a danger for their health. Thus, it is pivotal to provide simple and clear information and effective strategies for wearing a mask in order to intrinsic motivate the older adult population and counter any misinformation. Indeed, providing effective health communication is a key factor in the current pandemic (Finset et al., 2020). Similarly, information, motivation and strategies are the fundaments of each kind of adherent behavior, according the well-known Three-Factor Model (Martin & DiMatteo, 2013). Thus, it is essential to provide older adults with proper and understandable knowledge, suggesting easy strategies to manage face masks and triggering their inner motivation.

Lastly, motivating a person is not possible if not considering her/his *Assumptions*. According to Leventhal’s Common Sense Model of self-Regulation (Leventhal et al., 1980), people actively partake in the management of their health, putting into action different coping modalities according to their own beliefs and understandings. These cognitive and emotional representations, derived from internal (e.g., past experiences) and external inputs (e.g., others’ opinions), guide behavioral responses. Thus, an older adult may misunderstand the importance of the face mask due to cognitive impairment or false beliefs and subjective concerns. For instance, an individual may consider deleterious to breath in the face mask for a long period of time because of carbon dioxide toxicity. These mental representations and expectations may decrease motivation and may nourish a dangerous vicious cycle of oppositional behaviors regarding the donning of the face mask. Therefore, it is important to assess the individual concerns and beliefs regarding the adoption of this personal protective equipment and to address possible wrong expectations by providing simple and clear information, as well as proper reassurances. Doing so, it is possible to emotionally support the individual, addressing misconceptions and fears which prevent the use of face masks.

## Conclusion

Paying attention to the aforementioned aspects may help healthcare professionals, caregivers and policymakers to detect different kinds of hindrances to the adoption of face masks by older adult population, in particular when a slight cognitive impairment is present. In this regard, the adoption of feasible approaches and effective strategies has relevant social and political implications as the correct protection behavior may drastically reduce contagion and healthcare costs. In this vein, the CO-MAsk acronym suggests a simple mental roadmap to promote the adequate use of personal protective equipment and to emotionally support the older population along those hard times of sanitary crisis. The educational poster may be used in the healthcare context to effectively promote their adherence to this personal protection equipment. This aspect is indeed pivotal in present time and in the near future as further waves of COVID-19 or other pandemics cannot be excluded and these unwelcome scenarios would threaten the sustainability of the healthcare and socio-political system.

## Supplementary Materials

For this article Figure 1 in italian language is available via PsychArhives (for access see Index of Supplementary Materials below).
